# Maternal prenatal and perinatal psychiatric hospitalizations and academic performance in adolescent offspring: a register-based, data linkage, cohort study

**DOI:** 10.1192/j.eurpsy.2023.255

**Published:** 2023-07-19

**Authors:** G. Ayano, B. Dachew, K. Betts, R. Alati

**Affiliations:** School of Population Health, Curtin University, Perth, Australia

## Abstract

**Introduction:**

To the best of our knowledge, this is the first register-based cohort study to examine the association between maternal psychiatric hospitalizations before, during, and after pregnancy and the risk of lower academic performance in their adolescent children.

**Objectives:**

To investigate the risk of lower academic attainment in adolescent offspring of mothers with psychiatric hospitalizations before, during, and after pregnancy.

**Methods:**

This administrative health data-based cohort study used linked data obtained from health and educational registries in New South Wales, Australia (n=168, 528). Maternal psychiatric diagnosis before, during, and after pregnancy was measured by using ICD-10. The educational performance of the offspring was assessed by National Assessment Program for Literacy and Numeracy (NAPLAN). A multiple Logistic regression model was employed to investigate the association

**Results:**

After controlling for relevant covariates, we found that adolescent children of mothers with psychiatric hospitalizations before, during, and after pregnancy were at increased risk of substandard academic performance in all domains, with the highest odds for numeracy [OR, 2.88 (95%CI 2.50-3.31)] followed by reading [OR, 2.08 (95%CI 1.81-2.38)], spelling [OR, 1.74 (95%CI 1.51-2.01), and writing [OR, 1.56 (95%CI 1.34-1.80). In our sex-stratified analysis, maternal psychiatric hospitalizations demonstrated a stronger impact on the academic performance of females in all academic domains. Severe psychiatric disorders showed greater effects when compared to other psychiatric disorders.

**Conclusions:**

Early intervention strategies that aim to enhance academic performance in the children of mothers with psychiatric diagnoses before, during, and after pregnancy are needed.

**Disclosure of Interest:**

None Declared

